# Truncation of the unique N-terminal domain improved the thermos-stability and specific activity of alkaline α-amylase Amy703

**DOI:** 10.1038/srep22465

**Published:** 2016-03-01

**Authors:** Zhenghui Lu, Qinhong Wang, Sijing Jiang, Guimin Zhang, Yanhe Ma

**Affiliations:** 1 Hubei Collaborative Innovation Center for Green Transformation of Bio-Resources, The College of Life Science, Hubei University, Wuhan, 430062, People’s Republic of China; 2 Tianjin Institutes of Industrial Biotechnology, Chinese Academy of Science, Tianjin, 300308, China

## Abstract

High pH condition is of special interest for the potential applications of alkaline α-amylase in textile and detergent industries. Thus, there is a continuous demand to improve the amylase’s properties to meet the requirements set by specific applications. Here we reported the systematic study of modular domain engineering to improve the specific activity and stability of the alkaline α-amylase from *Bacillus pseudofirmus* 703. The specific activity of the N-terminal domain truncated mutant (N-Amy) increased by ~35-fold with a significantly improved thermo-stability. Kinetic analysis demonstrated that the *K*_*cat*_ and *K*_*cat*_/*K*_*m*_of N-Amy were enhanced by 1300-fold and 425.7-fold, respectively, representing the largest catalytic activity improvement of the engineered α-amylases through the methods of domain deletion, fusion or swapping. In addition, different from the wild-type Amy703, no exogenous Ca^2+^ were required for N-Amy to maintain its full catalytic activity, implying its superior potential for many industrial processes. Circular dichroism analysis and structure modeling revealed that the increased compactness and α-helical content were the main contributors for the improved thermo-stability of N-Amy, while the improved catalytic efficiency was mainly attributed by the increased conformational flexibility around the active center.

α-Amylase (1, 4-α-D-glucan glucanohydrolase; EC 3.2.1.1) belongs to glycoside hydrolase family 13 (GH13), catalyzing the endohydrolysis of α-D-(1–4) glucosidic linkages in starch and related polysaccharides with retention of the α-anomeric configuration in products^1^. It is generally accepted that the α-amylase family proteins hydrolyze the internal α-(1, 4) glucosidic linkages through α-retaining double displacement mechanism^2^.

α-Amylases are of ubiquitous occurrence and hold the maximum market share of enzyme sales with their extensive usage in starch industry and bakery^3^. Alkaline α-amylases obtain their maximum stability and activity under high pH conditions, which are of special interest for their potential applications in the textile industries and as ingredients of detergents. Due to conditions prevailing in the industrial applications are rather extreme, there is a continuing demand to screen novel α-amylases, or improve the activity and stability of amylases to meet the requirements set by specific applications^4^. For this purpose, alkaline and thermostable α-amylases from extremophiles were consecutively reported^5^, and protein engineering like rational design and directed evolution have been applied successfully to improve amylases’ characteristics^6^,^7^.

The typical α-amylase family proteins have four highly conserved regions, including the substrate-binding and catalytic sites, and three relatively conserved regions^8^. Structurally, all α-amylases possess a characteristic catalytic (α/β)_8_ or TIM barrel domain, A-domain, which contains the highly conserved residues involved in catalysis and substrate binding^9^. The α-amylase family proteins also have a B-domain, which is a large loop that protrudes between β-strand 3 and helix 3 in the (α/β)_8_-structure. The B- domain was reported to have an impact on the enzymes’ function and stability^10^. In most α-amylases, but not all, A- domain occurs at the N-terminal end of the protein^11^. Several other α-amylases were reported to have an extra N-terminal domain outside of the catalytic A- domain^11^, while the functional role of this domain on the enzyme is as yet uncertain.

Most extant proteins have modular design with independently stable subdomains^12^, thus, structure domain engineering including domain deletion, fusion or swap, were used to improve the enzymatic properties^13^. Wang *et al.* reported that truncation of the C-terminal CBD of endo-β-glucanase Egl499 improved its half-life at 65 °C, from 10 to 29 min^14^. The thermostability of amylopullulanase gt-apu from *Geobacillus thermoleovorans* NP33 was also enhanced after deletion of the N1 domain, which was contributed to the reduced structural flexibility in the noncatalytic region^15^. These evidences indicated that structure domain engineering is probably a good strategy superior to directed evolution and rational design for proteins with unknown 3-dimentional structures and function, especially lacking the suitable high-throughput screening methods.

In our previous study, an alkaline amylase Amy703 from *Bacillus pseudofirmus* 703 was successfully heterologously expressed in *Escherichia coli* BL21 (DE3) with enzymatic activity against soluble starch. Phylogenetic analysis demonstrated that Amy703 belongs to a new clade of glycoside hydrolase family 13 (GH13), and amino acid sequence analysis suggested that Amy703 contains a unique N-terminal domain, which combined together to identify Amy703 as a new alkaline amylase^16^. In this study, structure domain engineering strategy was used to improve the enzymatic properties of Amy703. Specifically, the mutants containing the N-terminal domain-truncation (N-Amy), C-terminal domain-truncation (C-Amy), and both terminal domains-truncation ((N+C)-Amy) were constructed and analyzed, respectively. Enzymatic characterization of these three purified mutants showed that the specific activity and thermo-stability of N-Amy was improved significantly compared to that of wild-type Amy703. Ca^2+^ -dependence property and substrate specificity of N-Amy were also altered. These results demonstrated that the structure domain engineering was useful to improve the specific activity and catalytic efficiency of Amy703. In addition, the impact of the unique N-terminal domain on the Amy703 was discussed as well.

## Materials and Methods

### Bacterial strains, plasmid and reagents

The expression plasmid pET28a*-Amy703* that carries the 2586-bp alkaline α-amylase gene, *Amy703*^16^, was used as the DNA template for structure domain deletion experiments. *E. coli* XL10-Gold and BL21 (DE3) were used as the cloning host and expression host, respectively. The synthesis of DNA primers and DNA sequencing were performed by GenScript Co. Ltd (Nanjing, China). Restriction enzymes, ExTaq DNA polymerase, T_4_ DNA ligase, and other enzymes used in the research were purchased from TakaRa (Dalian, China). All chemicals and reagents were analytical grade and commercially available.

### Homologous modeling of the tertiary structure of Amy703, N-Amy and C-Amy

The tertiary structures of wild-type Amy703 and mutants N-Amy and C-Amy were simulated using Molecular Operation Environment (MOE) software system. The template searching for homology modeling was performed by the MOE-Search PDB application, and the most suitable single template for homology modeling was chosen based upon full multiple alignments and Z-score significance testing. The stereochemical qualities of predicted structures were assessed from Ramachandran plots, and Energy Minimize was performed for residues that were beyond the acceptable phi/psi ranges.

### Construction of the expression plasmids

DNA primers were listed in Table 1. Primers P1 and P2 were used to amplify the coding region of N-Amy (truncated the N-terminal 200 amino acid residues). Primers P3 and P4 were used to amplify the coding region of C-Amy (truncated the C-terminal 84 amino acid residues). Primers P1 and P4 were used to amplify the coding region of (N+C)-Amy. Primers P1 and P5 were used to amplify the coding region of N-terminal domain. After being digested with *Bam*H I and *Xho* I, the different PCR products were cloned into the expression vector pET28a, which was digested with the same enzymes, to produce pET28a-N-Amy, pET28a-C-Amy, pET28a-(N+C)-Amy and pET28a-N-domain, respectively. The recombinant plasmids were confirmed by restriction enzyme digestion and sequencing.

### Expression and purification of mutants

*E. coli* BL21 (DE3) harboring recombinant plasmids were cultured in Luria Bertani (LB) medium containing 50 μg/ml kanamycin at 37 °C and 200 rpm till the OD_600_ reached to 0.6. Then isopropyl-β-d-thiogalactoside (IPTG) was added to a final concentration of 0.5 mM to induce the expression of enzymes. Cultivation was continuously grown for another 12 h at 18 °C followed by cells harvest. The harvested cell pellets were washed with PBS buffer and disrupted by sonication. Then the soluble fractions of the cell lysate were collected by centrifugation at 4 °C and 12 000 rpm for 20 min. The supernatant was subjected to a 5 ml HisTrap^TM^ FF column (GE Healthcare) which was pre-equilibrated with 50 mM Tris-HCl (pH 9.0) containing 0.5 M NaCl and 30 mM imidazole. After being extensively washed with the same buffer, the binding proteins were eluted with 50 mM Tris-HCl (pH 9.0) containing 0.5 M NaCl and 300 mM imidazole. The desalting of the eluent was performed through a HiTrap^TM^ Desalting column (GE Healthcare) equilibrated with 50 mM Tris-HCl (pH 9.0). The molecular weight and homogeneity of the purified proteins were evaluated by SDS-PAGE, and the protein concentration was determined by Bradford assay using bovine serum albumin (BSA) as the standard^17^.

### Measurement of enzymatic activity and kinetic parameters

The α-amylase activity was assayed by measuring the amount of reducing sugar released in the enzymatic hydrolysis reactions using 1% (w/v) soluble starch (Sigma) as substrate^16^. One unit of α-amylase (U) was defined as the amount of enzyme of releasing 1 μmol of reducing sugar per minute under the assay conditions, and glucose was used as a standard. The specific activity (U/mg) is the moles of product formed by an enzyme in a given amount of time under given conditions per milligram of total protein. The kinetic parameters of mutant N-Amy were assayed in Tris-HCl buffer (pH 9.5, 50 mM) at 50 °C. The *K*_m_ and *V*_max_ were estimated from Eadie-Hofstee plots.

### Characterization of biochemical properties

Optimal reaction temperature was determined at various temperatures ranging from 30 °C to 55 °C in 50 mM Tris-HCl buffer (pH 9.0). Thermal stability was evaluated by pre-incubating the enzyme for 20 min under specific conditions (30 °C to 50 °C), followed by residual activities measurement at pH 9.5 and 50 °C.

The optimal pH for N-Amy703’s enzymatic activity was assayed by different buffers with different pH values, including 50 mM Tris-HCl buffer (pH 7.5–9.0) and 50 mM glycine-NaOH buffer (pH 9.0–10.0). The pH stability was evaluated by pre-incubating the enzyme in buffers with pH values from 7.5 to 10 at 4 °C for 12 h, followed by residual activities measurement at pH 9.5 and 50 °C.

The effects of 10 mM various metal ions on the activity of N-Amy were determined under optimal conditions, which were normalized to the enzyme without any addition of metal ions.

The substrate specificity of purified N-Amy was assayed at 50 °C with 1% (w/v) of soluble starch, amylose from potato, amylopectin from maize, α-cyclodextrin, pullulan, and glycogen from rat liver (all purchased from Sigma) in Tris-HCl buffer (50 mM, pH 9.5). The amount of reducing sugar produced was measured as mentioned above.

### Circular dichroism (CD) analyses

Circular dichroism (CD) spectra of Amy703 and N-Amy were measured with a 1-cm path-length quartz cuvette at a protein concentration of 0.1 mg/ml in glycine-NaOH buffer (pH 9.0, 20 mM). The spectropolarimeter and xenon lamp were warmed up for at least 30 min prior to experiments to minimize baseline signal drift. Ellipticity data were collected between 190 and 260 nm and the spectrum of a buffer blank was subtracted. The lengths and fractions of α-helixes and β-sheets were determined with an online analysis of CD data using DichroWeb^18^. The melting temperature (T_m_) of Amy703 and N-Amy were also determined using CD, in which the temperature gradually increased from 30 °C to 65 °C at 0.5 °C/min.

### Substrate binding assay

Purified Amy703 and N-Amy (15–30 μg) were mixed with 5 mg amylose (almost insouble, from potato, Sigma) in 50 mM Tris-HCl buffer (pH 9.0). After being gently shaked at 30 °C for 1 h, the binding mixtures were centrifuged at 12,000 rpm for 5 min. The amounts of unbound enzymes in the supernatant were detected by SDS-PAGE with the binding mixtures without amylose as the control.

## Results

### Structure-based domain truncation mutation

**Figure 1:**
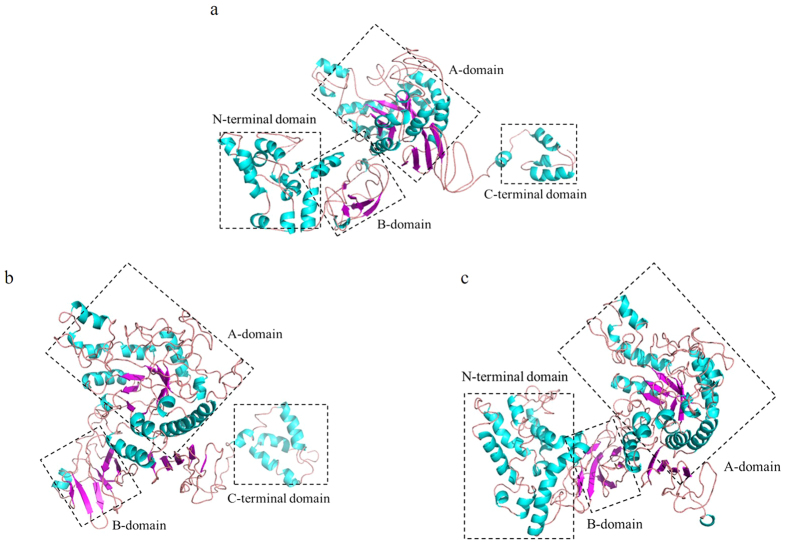
The tertiary structure of Amy703 (**a**), N-Amy (**b**) and C-Amy (**c**). The α-helixes and β-sheet are shown in blue and purple, respectively. The structure domains are boxed in black dots.

The tertiary structure of Amy703 was modeled by MOE software, and the maltogenic amylase with PDB number of 1SMA. A was chosen as the structural model to simulate the pseudo-tertiary structure Amy703. The simulated structure indicated that Amy703 consisted of four modular domains, including N-terminal domain (Gln1-Lys200), B-domain, A-domain, and C-terminal domain (Val785-Lys862) (Fig. 1). The A-domain and B-domain constituted the core catalytic domains of Amy703, which were reported to play an essential role in maintaining the activity of α-amylases^19^. With the B-domain and A-domain unchanged, three mutants with either N-terminal domain or C-terminal domain deletion or both domains deletion were constructed to explore the effects of these two domains against the Amy703 activity. The (N+C)-Amy mutant kept the core catalytic domains untouched, covering the Asn201-His784 region, and the N-Amy mutant and the C-Amy mutant lacked the N-terminal domain (Gln1-Lys200) and the C-terminal domain (Val785-Lys862), respectively.

### Expression and purification of the mutants

The N-Amy, C-Amy, and (N+C)-Amy mutants were anchored with 6xHis tag at N-terminus followed by heterologously expressed in *E. coli* BL21 (DE3) cells. The recombinant proteins were then purified using Ni-NTA resin, and characterized for their activities against different substrates. The specific activity of N-Amy (170 U/mg) was significantly improved, exhibiting a proximate 35-fold higher activity against soluble starch than that of wild-type Amy703 (4.9 U/mg). The specific activities of the C-Amy and (N+C)-Amy were 0.48 U/mg and 1.02 U/mg, respectively, presenting an obvious decrease compared to the wild-type Amy703. These combined results suggested that the C-terminal domain could up-regulate the activity of Amy703 against soluble starch, while N-terminal domain probably had down-regulation effects. In addition, the (N+C)-Amy exhibited similar specific activity with the C-Amy, confirming the fact that the domain A and domain B were mainly responsible for the catalytic activity of Amy703.

### Effects of temperature and pH on the activity and stability of N-Amy

**Figure 2:**
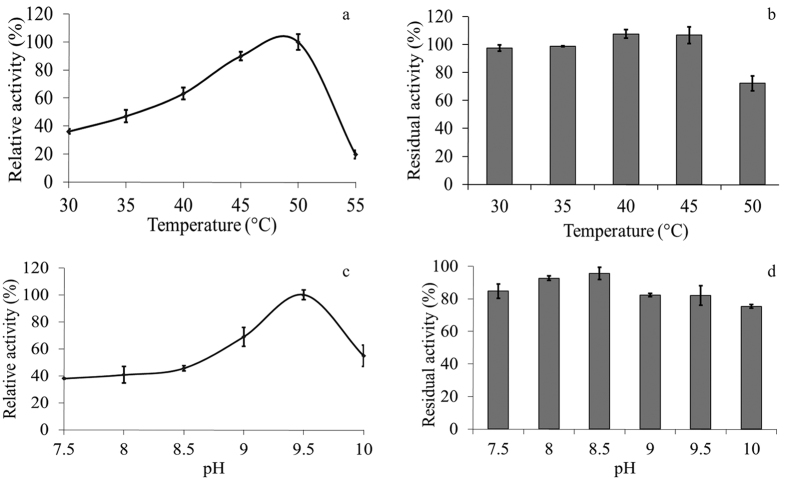
Effects of temperature and pH on the activity and stability of N-Amy. (**a)**, Effects of temperature on the activity of N-Amy. The reaction was conducted from 30 to 55 °C in 50 mM Tris–HCl buffer (pH 9.0). **(b)**, Effects of temperature on the stability of N-Amy. The thermal stability of N-Amy was determined at the indicated temperature in 50 mM Tris–HCl buffer (pH 9.0) for 20 min. After incubation, the residual activity of enzyme was measured at pH 9.5 and 50 °C. Each value of the assay was the arithmetic mean of triplicate measurements. **(c)**, Effects of pH on the activity of N-Amy. The reaction was conducted at 50 °C with soluble starch as substrate in different buffers: 50 mM Tris–HCl (pH 7.5–9.0) and 50 mM glycine–NaOH (pH 9.0–10.5). **(d)**, Effects of pH on the stability of N-Amy. The stability was determined at different pH values ranging from 8.0 to 10.5 (50 mM Tris–HCl or 50 mM glycine–NaOH) at 4 °C for 12 h. The residual activity was measured at pH 9.5 and 50 °C. Each value of the assay was the arithmetic mean of triplicate measurements.

The detailed biochemical properties of the N-Amy were further evaluated, as the specific activity of this mutant was significantly improved. The optimum temperature of N-Amy was observed at 50 °C (Fig. 2a), about 10 °C higher compared to that of Amy703^16^. Besides, the activity of N-Amy retained above 75% after 20 min incubation at 50 °C (Fig. 2b), while only 10% activity was retained for Amy703 under the same condition^16^. Obviously, the thermostability was remarkably enhanced when the N-terminal domain was removed in Amy703.

Comparing to the wild-type Amy703 which presented the highest activity at pH 9.0^16^, the optimum pH of N-Amy was shifted to 9.5 (Fig. 2c). More importantly, the N-Amy remained 60% relative activity at pH 9 and 10, indicating a favorable property for its application in alkaline conditions. As shown in Fig. 2d, N-Amy maintained high stability over a wide range of pH. Further evidence suggested that above 80% of its maximal activity was retained after incubation at 4 °C in pH 7.5–10 for 12 h, which was consistent with the wild-type Amy703.

### Effects of various metal ions on the activity of N-Amy

Many amylases are metal dependent enzymes, including the Amy703^16^. The effects of different metal ions on the activity of N-Amy were also assayed to evaluate if the N-terminal domain would affect the Amy703’s metal ion binding ability (Table 2). The results indicated that 10 mM EDTA almost abolished the activity of N-Amy, suggesting that metal ion(s) was still required for N-Amy to maintain its activity. But surprisingly, 10 mM Ca^2+^ inhibited the activity of N-Amy that only 59.4% relative activity was retained. This finding was completely opposite to the previous study, in which the activity of the Ca^2+^-dependent wild-type Amy703 was significantly enhanced by 300% at the presence of 10 mM Ca^2+^ 16. To further analyze the effect of Ca^2+^ on N-Amy, 10 mM CaCl_2_ was added into enzyme solution that was premixed with 10 mM EDTA. Interestingly, the activity of N-Amy was almost fully recovered, demonstrating that Ca^2+^ was still required to maintain the activity of N-Amy. Mg^2+^ was previously also found to dramatically enhance the activity of Amy703, while no obvious improvement for N-Amy was observed. On the other hand, Na^+^, K^+^ and Li^+^ inhibited the wild-type Amy703, but slightly improved the activity of N-Amy. Same with Amy703, Hg^2+^ and Ni^2+^ were also strong inhibitors for N-Amy with relative activity decreased to 2%.

### The kinetic parameters of N-Amy

The *K*_*m*_ and *V*_*max*_ values of N-Amy on soluble starch were measured from Eadie-Hofstee plots. N-Amy hydrolyzed soluble starch with *K*_*m*_ of 11.9 mg/ml, which was higher than that of Amy703 (3.9 mg/ml)^16^. The *V*_*max*_ of N-Amy (1.32 μmol/min) was considerably higher than that of Amy703 (17.7 nmol/min/mg). The catalytic constant (*K*_*cat*_) and catalytic efficiency (*K*_*cat*_/*K*_*m*_) of N-Amy were measured as 50.2 × 10^3^/min and 4.3 × 10^3^ ml/mg/min, respectively. These kinetic parameters for the wild-type Amy703 were 39.4/min and 10.1 ml/mg/min, respectively, indicating a 425.7-fold enhancement of *K*_*cat*_/*K*_*m*_. These results indicated that the deletion of the N-terminal domain could dramatically enhance the catalytic efficiency of Amy703. These results indicated that truncating the N-terminal domain of Amy703 reduced its binding for substrate, while dramatically enhanced its catalytic efficiency.

### The analysis of structure variation of N-Amy and C-Amy

**Figure 3:**
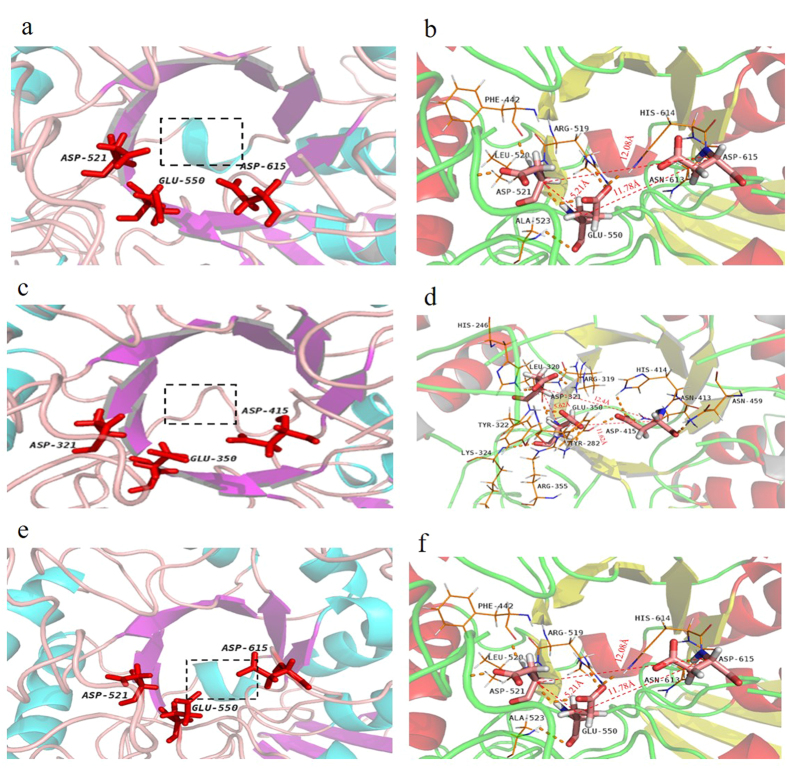
The detailed structure around the active sites of Amy703, N-Amy and C-Amy. The changes of conformational flexibility around the active sites of Amy703, N-Amy and C-Amy were shown by **(a,c,e)**, respectively. The amino acids of active sites were labeled by red sticks. The peptide Trp331-Asp334 is boxed by black dots. The changes in structure around the catalytic triad of Amy703, N-Amy and C-Amy were shown by **(b**,**d**,**f)**. The amino acids of active sites were labeled by colored sticks. The lines are residues forming hydrogen bonds with the catalytic triad. The orange dotted line is the hydrogen bond. The red dotted line is the distance between the catalytic triad, and distances are shown by numbers.

To investigate the molecular mechanism for the improved activity of N-Amy, the 3-D structure of N-Amy and C-Amy, which was treated as the second control for the comparison, were simulated using MOE (Fig. 1), followed by the computational analysis of the conformational flexibility around the active sites and distances among active sites (Fig. 3). Compared to the simulated structure of Amy703 and C-Amy, the structural flexibility around the active center of N-Amy was significantly increased, which was annotated by change of alpha helix in Amy703 and C-Amy to loop of residues Trp331-Asp334 (Fig. 3a,c,d), corresponding to Trp131-Asp134 of N-Amy. In addition, deletion of the N-terminal domain or C-terminal domain of Amy703 altered the distances between the catalytic triad, increasing the distances between Asp321 and Asp415 in N-Amy (corresponding to Asp521 and Asp615 in C-Amy) from 11.44 Å to 12.4 Å and 12.08 Å, respectively. The distances between Glu350 and Asp415 in N-Amy (corresponding to Glu550 and Asp615 in C-Amy) were also increased compared to that in Amy703, from 9.65 Å to11.62 Å and 11.78 Å respectively (Fig. 3b,d,f). Accordingly, the hydrogen bonds of the catalytic triad were altered after domain deletion. For example, Glu550 formed hydrogen bonds with L520, Y522, A523 and S554 in Amy703, while it formed hydrogen bonds with L320, Y322, K324 and R355 in N-Amy (Fig. 3b,d). C-terminal domain deletion also partially breaks the TIM barrel fold, in which the strands β7 and β8 in TIM barrel of Amy703 turn into loop structure (Fig. 3e,f).

### The CD analysis of N-Amy and Amy703

**Figure 4:**
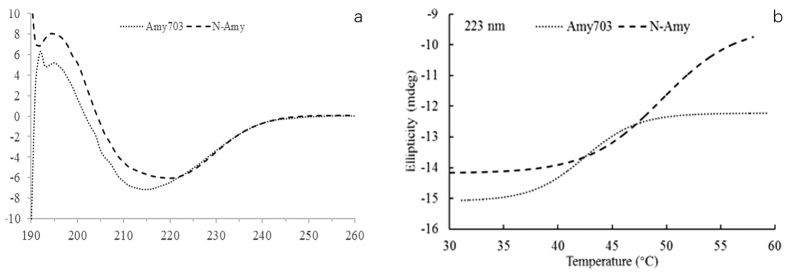
CD analysis of Amy703 and N-Amy. (**a)** is the Far-UV CD spectra of Amy703 and N-Amy. **(b)** is the melting temperature (T_m_) of Amy703 and N-Amy.

To evaluate the structural change introduced by the N-terminal domain to the Amy703, the secondary structure contents of Amy703 and N-Amy were experimentally determined using far UV-CD spectrum (190 nm–260 nm) analysis. As shown in Fig. 4a, the secondary structures were significantly altered after N-terminal domain deletion, the α-helical component of N-Amy was increased from 33.7% to 45.1%, which may contribute to the stabilization of N-Amy. Meanwhile, the melting temperature (*T*_m_) of N-Amy was 48.6 °C, increased by 7 °C compared to Amy703 (Fig. 4b). Both explained the reason of higher thermo-stability of N-Amy.

### The function analysis of N-terminal modular domain and C-terminal modular domain

**Figure 5:**
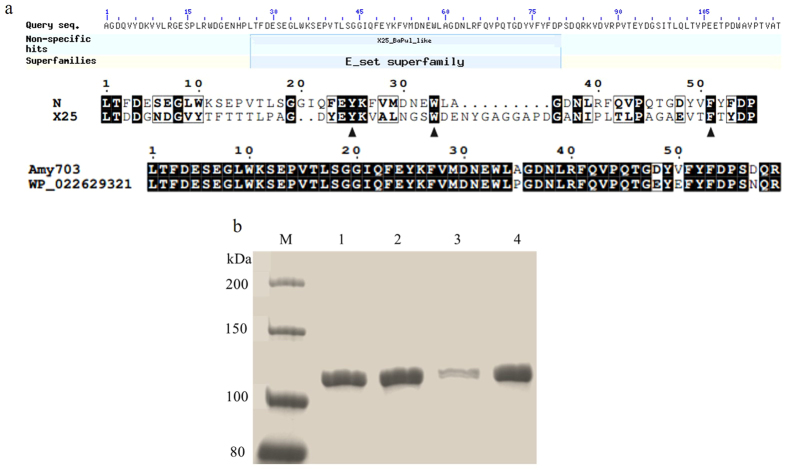
The function of N-terminal modular domain. (**a**), The BLAST analysis of N-terminal modular domain. WP_022629321.1: alpha-amylase from *Bacillus marmarensis*. (**b**), The binding assays of Amy703 and N-Amy to amylose. Purified Amy703 or N-Amy was mixed with or without amylose in 50 mM Tris-HCl buffer at 30 °C for 1 h. After centrifugation at 12,000 rpm, unbound proteins in supernatant were detected by 10% SDS–PAGE. 1, N-Amy with amylose. 2, N-Amy with amylose as a control. 3, Amy703 with amylose. 4, Amy703 without amylose as a control. All the experiments were performed under the same conditions with SDS-PAGE results being cropped for clear presentation.

Very few α-amylases with the N-terminal domain preceding the catalytic domain A have been reported so far, therefore, the function of the unique N-terminal domain of Amy703 was investigated. Sequence analysis of the N-terminal structure domain of Amy703 (Gln1-Lys200) was performed against the Conserved Domain Database (CDD)^20^, revealing that partial region (Leu26-Pro80) of the N-terminal domain belonged to the conserved *E*_set superfamily (Fig. 5a). As the *E*_set superfamily was known as an adaptor domain related to the carbohydrate substrate binding in some catalytic enzymes^21^, whether the N-terminal domain was involved in the binding of starch was investigated using insoluble amylose binding assays. After incubation with amylose at 30 °C for 1 h, the amounts of Amy703 and N-Amy in the supernatant were detected by SDS-PAGE, in which decreased protein amount in the supernatant indicated a strong binding of amylose. The residual Amy703 in the supernatant remarkably decreased compared with the control (Fig. 5b), while no detectable change of N-Amy in the supernatant was observed between binding mixture with and without amylose (Fig. 5b). These observations demonstrated that N-terminal domain of Amy703 was involved in binding of amylose, according with the substrate specificity analysis, which indicated that only Amy703 displayed detectable hydrolytic activity toward amylose and amylopectin. The involvement of the N-terminal domain in the recognition of Amy703 against amylose also suggested that it may be involved in the recognition of mixed alpha-1, 6/alpha-1, 4 linked D-glucan polysaccharides (amylopectin) like X25 domain of *B. acidopullulyticus* pullulanase^22^.

The possible function of C-terminal domain was also investigated by sequence analysis. The result showed that C-terminal domain does not belong to any conserved domain (see Supplementary Fig. S1 online). Because truncation of the C-terminal domain partially broken the TIM barrel structure, we speculate that C-terminal domain may directly interact with the core catalytic domain, and plays an essential role in maintaining the structural stability of Amy703.

## Discussion

Enzymes’ properties have been successfully modified to fulfill requirements of industrial applications by various protein-engineering strategies^23^,^24^,^25^. Enzymes typically have two functional units, an active center which catalyze the reaction and a scaffold in which the active center was inserted^26^. As each structure domain of proteins can normally be independently stable and folded, structure domain engineering including domain deletion, fusion or swap would be an alternative to engineering enzymes characteristics, which will alter protein scaffold but still hold the active center in place. In fact, as a widely used enzyme in industries, several studies have been reported to improve the catalytic activity of α-amylases by domain deletion or fusion^14^,^15^. In the present study, the first systematic study of modular domain engineering was reported to improve the specific activity and stability of Amy703. Characterization of the constructed mutants indicated that the N-terminal domain deletion mutant (N-Amy) has a much higher specific activity (~35-fold) than that of wild-type Amy703, representing the largest improvement in catalytic activity of the engineered α-amylases by domain deletion, fusion or swap that has been reported. In addition, it is worthy to note that the half-life of N-Amy at 50 °C was extended 4 folds, and the melting temperature was increased from 41.4 °C to 48.6 °C, expanding its application in extreme conditions.

To understand the structural alteration responsible for the improved properties of N-Amy, comparative evaluate structure model of Amy703, C-Amy and N-Amy703 was performed (Fig. 3). The distances between the catalytic triad are both increased in N-Amy and C-Amy (Fig. 3b,d,f), generating a much opener catalytic cleft for binding substrate. Different from C-Amy, the secondary structure of peptide Trp331-Asp334 in N-Amy was changed from alpha helix to loop, increasing the conformational flexibility around the active center after N-terminal domain deletion (Fig. 3a,c,d). The enhanced flexibility around the active center of N-Amy may decrease the steric hindrance for substrates entry or products release, thus increasing the catalytic efficiency of Amy703. The similar mechanisms were also reported in other enzymes^27^. While truncation of the C-terminal domain partially breaks the TIM barrel structure (Fig. 3e,f), which contain amino acids that are involved in catalysis and substrate binding. This partially broken TIM barrel structure may be mainly responsible for the significantly low activity of C-Amy and for the increased distances between the catalytic triad. Therefore, we speculate that the C-terminal domain may directly interact with the core catalytic domain, and play an essential role in maintaining the structural stability of Amy703. The UV-CD spectrum (190nm–260nm) analysis indicated that the N-terminal domain truncation arouse the structural alteration of the remaining protein, causing the α-helical component in N-Amy being significantly increased to 45.1%. The thermo-stability of proteins is the result of the combination of several factors acting in synergistic manner^28^. It is known that the alpha helix plays an important role in proteins’ thermo-stability, and the break of contacts between neighboring residues in an α-helix is one of the most energy-consuming part during protein denaturation^29^. The increased α-helical component is probably a contributing factor for the enhanced thermo-stability of N-Amy. Besides, the deletion of N-terminal domain (200 amino acids) may also contribute the thermo-stability of N-Amy by improving its compactness. This correlates to the previous report that the shortening of domains can contribute to compactness of proteins, improving the protein thermo-stability^30^.

It is a paradox between calcium requirement for catalytic activity of N-Amy and its inhibitory effect when added in molar excess. α-Amylase PWA from *Pyrococcus woesei* was reported to has a similar property that zinc has positive and negative effect on its activity in a concentration-dependent manner^31^. The structure of PWA reveals an activating two-metal (Ca, Zn) binding site and a second inhibitory zinc binding site^31^. According to the effects of Ca^2+^ on the activity of Amy703 and N-Amy, we speculate two Ca^2+^ binding sites existing in Amy703. The first Ca^2+^ binding site has a higher affinity of Ca^2+^, which is critical to the enzyme activity. It is speculated that the binding of Ca^2+^ to this site affects the enzyme structure, resulting in its active conformation. Therefore, Ca^2+^ chelation caused by EDTA can abolish the enzyme activity. Comparably, the second Ca^2+^ binding site has a lower affinity of Ca^2+^, which is more related to the enzyme stability. Hence, the exogenously added Ca^2+^ will bind to this site to stabilize the structure of Amy703, so the enzyme activity is boosted. Other than Amy703, N-Amy possesses a different metal control mechanism due to its different structure caused by the N-terminal domain deletion. It is speculated that the binding of Ca^2+^ to the second Ca^2+^ binding site in N-Amy destabilizes the structure of N-Amy, causing the inhibiting effects. In the absence of exogenous Ca^2+^, the first Ca^2+^ binding site, which has a higher affinity for Ca^2+^, binds Ca^2+^ and display hydrolytic activity. However, when exogenous Ca^2+^ (10 mM) is added, the second Ca^2+^ bind site will bind the excessed Ca^2+^ to prompt the inhibitory effect against N-Amy. This inhibitory mechanism of N-Amy leads to the results that addition of 10 mM CaCl_2_ without EDTA will inhibit the enzyme activity, while addition of 10 mM CaCl_2_ premixed with 10 mM EDTA will fully recover the enzyme activity. Namely, Ca^2+^ has both positive and negative effects on the activity of N-Amy in a concentration-dependent manner. The property that does not require the addition of metal ions for activity makes N-Amy exceed most other α-amylases for many industrial processes, where the added Ca^2+^ must be removed from the product streams.

The starch-processing enzymes carrying N domain are new types of amylase, in which the function of the unique N-terminal domain is unclear. Sequences analysis indicated that the N-terminal domain of Amy703 contains a conserved *E*_set superfamily, a subset of which was recently identified as member of the CBM48 (Carbohydrate Binding Module 48) family. In addition, this region was also suggested to be an X25_BaPul_like domain (non-specific hits). Interestingly, it was reported that the N1 domain of amylopullulanase gt-apu from *Geobacillus thermoleovorans* NP33 was also contains the X25_BaPul_like domain, whose deletion altered the substrate-specificity of gt-apu^15^. In here, amylose binding assays demonstrated that the N-terminal domain contributed the binding of Amy703 to amylose (Fig. 5). All these combined demonstrated that N-terminal domain is probably a starch binding domain in Amy703.

From an evolutionary perspective, the N-terminal domain should have special function for Amy703 to compensate the sacrifice of activity and thermal stability. Substrate specificity analysis suggested that Amy703 was hydrolytically active on substrates such as soluble starch, amylopectin from potato, and amylose from potato. In contrast, N-Amy can only recognize soluble starch (Fig. 5), while exhibited 35-fold higher hydrolytical activity against soluble starch compared to Amy703. It can be speculated that the unique N-terminal domain is obtained by the adaptive evolution of Amy703. *B. pseudofimus* 703 retained this N-terminal domain, though detrimental to activity and stability of Amy703, to expand the available substrate spectrum and to live in different niches.

In conclusion, a simple and universal protein engineering strategy was described to reconstruct modular enzymes, and verified of its effectiveness by deleting the modular domain of Amy703. It is the first report that N-terminal domain deletion of amylase not only improved significantly its thermos-stability and activity but also changed the Ca^2+^ -dependent activity. Moreover, the N-terminal domain of Amy703 also contributed to its substrate binding ability.

## Additional Information

**How to cite this article**: Lu, Z. *et al.* Truncation of the unique N-terminal domain improved the thermos-stability and specific activity of alkaline α-amylase Amy703. *Sci. Rep.* **6**, 22465; doi: 10.1038/srep22465 (2016).

## Supplementary Material

Supplementary Figure S1

## Figures and Tables

**Table 1:** Primers used in this study.

Name	Sequence (5′-3′)
P1	CGCGGATCCAATGTCTCTCACAACTTTAACCACAACC
P2	CCGCTCGAGTTATTTCTGACCTCGCTTGTCACTC
P3	CGCGGATCCGCAGGAGACCAAGTATATGATAAAGTCG
P4	CCGCTCGAGTTAATGTTCAATGTCCTTTTCTTCCTCA
P5	CCGCTCGAGTTTAGCCACAGGAAGGCCTGTC

**Table 2:** Effects of metal ions on the activity of N-Amy.

Metal ions (10 mM)	Relative activity (%)
No addition	100
Na^+^(NaCl)	122.2
Ca^2+^(CaCl_2_)	59.4
Mg^2+^(MgCl_2_)	109
Ni^2+^(NiCl_2_)	1
Li^+^(LiCl)	134.6
K^+^(KCl)	119.7
Co^2+^(CoCl_2_)	34.2
Hg^2+^(HgCl_2_)	2.1
EDTA	0.3

The assay was taken at optimal conditions (50 °C, pH 9.5) with soluble starch as substrate. No addition means the activity of N-Amy was determined in buffers without the addition of any ions.
