# Supplementary material for: Truncation of the unique N-terminal domain improved the thermos-stability and specific activity of alkaline α-amylase Amy703
Source: Sci Rep. 2016 Mar 1;6:22465. doi: 10.1038/srep22465 (PMC4772547; doi:10.1038/srep22465)

Manuscript tittle: Truncation of the unique N-terminal domain improved the thermos-stability and specific activity of alkaline α-amylase Amy703

Author list: Zhenghui Lu [1], Qinhong Wang [2], Sijing Jiang[1], Guimin Zhang[1*], Yanhe Ma[2]

Legend:

Supplementary Figure S1. The BLAST analysis of C-terminal domain of Amy703 against the Conserved Domain Database (CDD).

Supplementary Figure S1

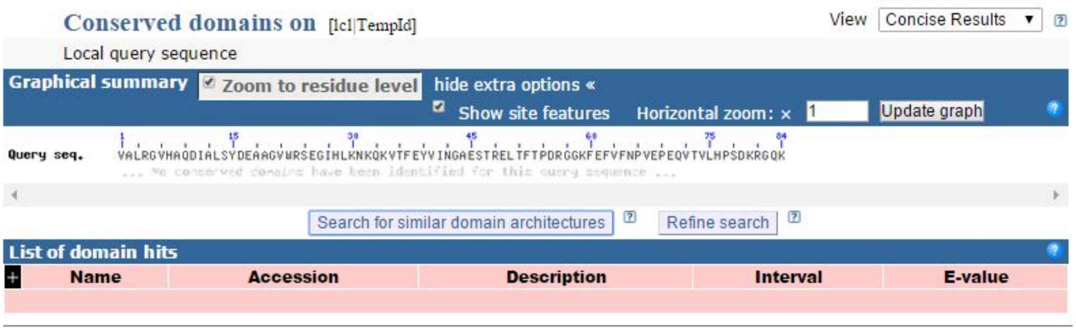

Supplement: Supplementary Figure S1 [file srep22465-s1.pdf]
